# Rush Shippen Huidekoper

**Published:** 1901-12

**Authors:** 


### EDITORIALS.

### RUSH SHIPPEN HUIDEKOPER.

A GREAT leader has fallen. The mantle of death has closed the earthly career of a strong man—strong in that which is best remembered of men's lives. His may be classed among those who are said to have led strenuous lives. Clothed by nature with great physical strength, remarkable will-power, great force of character, and endowed with a goodly share of worldly wealth; a man of the highest and best ideals in all that he elected to do; successful in a remarkable degree along lines that are too seldom counted as the highest winnings of the world. He touched nothing that he did not leave it better than he found it. In veterinary journalism he raised it to a higher plane; in college work he made it stronger and better than ever before; in State legislation he aimed always at the highest ideals of professionalism; in State Boards of Examiners he ever advocated a higher grade of work; in the field of association work he strove always to make loftier its aims and purposes; in his masterful leadership for recognition and rank of his profession in the Army he never forgot, in the battles he waged, the higher purposes to be gained for the true growth of his adopted profession. In all of these spheres of work as editor, teacher, officer, practitioner, examiner, and leader he gave lavishly of his time, liberally of his means, and spent unselfishly of his wonderful energy and indomitable courage in making successful, to a remarkable degree, these higher ideals that he ever held of what he singled out to do.

Unselfish in all that he did, frank and honest in all his dealings with his fellow-man, unresentful of any blows aimed to do him harm, and with the utmost confidence in all whom he met, such was the life of Rush Shippen Huidekoper, who has passed from our midst too soon.

